# Investigation of Mechanical and Magnetic Properties of Co-Based Amorphous Powders Obtained by Atomization

**DOI:** 10.3390/ma14237357

**Published:** 2021-11-30

**Authors:** Anna Kuś, Wirginia Pilarczyk, Aleksandra Małachowska, Andrzej Ambroziak, Piotr Gębara

**Affiliations:** 1 Faculty of Mechanical Enginerging, Wrocław Universtity of Science and Technology, Wyb. Wyspiańskiego 27, 50-370 Wrocław, Poland; aleksandra.malachowska@pwr.edu.pl (A.M.); andrzej.ambroziak@pwr.edu.pl (A.A.); 2 Faculty of Mechanical Engineering, Silesian University of Technology, ul. Akademicka 2A, 44-100 Gliwice, Poland; wirginia.pilarczyk@polsl.pl; 3 Department of Physics, Częstochowa Universtity of Technology, Armii Krajowej 19, 42-200 Częstochowa, Poland; piotr.gebara@pcz.pl

**Keywords:** co-based amorphous alloy, annealing, hardness, elastic module, magnetic properties, Mössbauer, amorphous alloy, metallic glass

## Abstract

Properties of Co-based alloys with high Glass Forming Ability (GFA) in the form of powder are still not widely known. However, powders of high GFA alloys are often used for the development of bulk metallic glasses by additive manufacturing. In this work Co_47.6_B_21.9_Fe_20.4_Si_5.1_Nb_5_% at. and Co_42_B_26.5_Fe_20_Ta_5.5_Si_5_Cu_1_% at. were developed by gas-atomization. Obtained powders in size 50–80 µm were annealed at T_g_ and T_x_ of each alloy. Then SEM observation, EDS analyses, differential thermal analysis, X-ray diffraction, nanoindentation, Mössbauer, and magnetic properties research was carried out for as-atomized and annealed states. The gas atomization method proved to be an efficient method for manufacturing Co-based metallic glasses. The obtained powder particles were spherical and chemically homogeneous. Annealing resulted in an increase of mechanical properties such as hardness and the elastic module of Co_47.6_B_21.9_Fe_20.4_Si_5.1_Nb_5_% at and Co_42_B_26.5_Fe_20_Ta_5.5_Si_5_Cu_1_%, which was caused by crystallization. The magnetic study shows that Co_47.6_B_21.9_Fe_20.4_Si_5.1_Nb_5_ and Co_42_B_26.5_Fe_20_Ta_5.5_Si_5_Cu_1_ are soft magnetic and semi-hard magnetic materials, respectively.

## 1. Introduction

Amorphous alloys are a specific group of metal alloys in which the structure of the atoms that build solid-state objects is arranged without repeating the long-range order [1,2]. To obtain that state, the melted alloy needs to be cooled at a speed higher than the critical cooling rate. This cooling stops dynamic atom diffusion and enables the development of an amorphous solid-state structure of a metal alloy [3].

**Figure 1:**
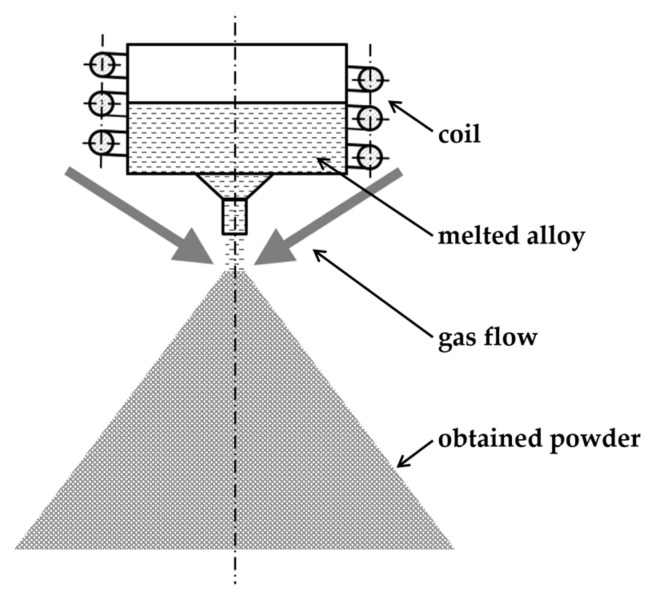
Schematic of the atomization process.

One of the methods that ensure cooling with a high enough speed is the gas atomization shown in Figure 1. Researchers [3,4,5] proved that atomization is a proper method to prepare amorphous alloys in a powder form. However, research shows that fully vitrification of powders depends on the size of obtained particles [6] and glass-forming ability of the processed alloy [7]. In article [8] researchers were able to simulate an atomization process for a Fe-based alloy with high glass forming ability. The simulation results of particle size distribution were confirmed by experimental results. Experimenters show a correlation between the pressure of the gas with particle size distribution. Obtained powders were fully amorphous for particle size 25–45 µm.

Gas atomization allows obtaining spherical and chemically homogeneous powder particles, which is essential to keep the high glass forming ability of the alloy. While new methods such as in-situ alloying of blended powder [9] is cost-effective, it often requires heat treatment to homogenize the chemical composition, which is unfavourable for metallic glasses.

Furthermore, powders obtained through atomization were successfully exploited in the production of bulk amorphous glasses by additive manufacturing. Ref. [10] reported efficiently produced samples by selective laser melting using gas-atomized powder (10–90 µm particles size) of Ti-based alloys with high glass-forming ability (critical casting thickness up to 7 mm). Remarkably, in [10] research did not obtain the amorphous structure of the alloy by atomization (powder form), while obtained SLM samples from that powder had an amorphous structure. In [11] for SLM Fe-based amorphous alloys were used in form of particles size 2–53 µm received by gas atomization. Like before, the powder was a particular crystal—the volume of the crystalline phase was 10.1%, but the parts of a developed sample were nearly almost amorphous.

Additive manufacturing is a technology also considered as a solution for the problem with critical raw materials. The latest review article [12] reports that additive manufacturing prevents developing waste through the production process and can change the whole chain that starts from suppling to distribution, which will occur in a much better situation of the critical raw materials market.

The atomization process is also in the interest of researchers to produce high entropy alloys [13,14], which shows that atomization is a process suitable for a wide range of materials and worth studying more precise for materials from different groups. Moreover, many powders developed through this process are suitable to use as a feedstock for additive manufacturing [15]. In this article, amorphous metals obtained thou atomization will be tested.

The amorphous state of the alloy provides properties different from those of classic alloys with crystal/polycrystal structure [16]. With that order of atoms, different from the ‘classic’ alloys used in the global market, amorphous alloys give new possibilities of obtaining material harder and more durable with a young module with higher, better anticorrosion properties, and enormous good magnetic properties. That interest in amorphous alloys started when the first amorphous alloy was established by Klement [17] in 1960 and has lasted until now [5,18].

The cobalt-based amorphous alloys investigated in this work have relatively good glass forming ability (GFA). In [19] CoMoPB rods achieve 4.5 mm of critical diameter, and in [20] even 5.0 mm for the CoFeNiBSiNb alloy. Other parameters related to GFA, namely, the supercooled liquid region (ΔT_x_) for some Co-based alloys can even reach 111 ± 5 °C [21]. This very good GFA of Co-based alloys allows different shape samples to be developed for research. As described above, the researchers were working on bulk metallic glasses (BMG) rods made using the copper mold injection casting method, but researchers also successfully obtained ribbons by melt spinning processes [22,23]. A more interesting shape, which was also studied in this work, is the powder form of an amorphous Co-based alloy. In [5] the researchers reported that they successfully obtained a powder of Co-based alloy by gas atomization, the same technology as used in this work. In addition, powder from [4] was used to develop an amorphous coating by laser cladding, so it is the next possibility of a form of Co-based alloys (amorphous coatings). However, researchers are still working on the BMG made by additive manufacturing. Although, successfully made by SLM BMG of other amorphous alloys and printing layers from Co-based alloys (laser cladding) implies that printing BMG Co-based amorphous alloys is possible.

Amorphous alloys based on Cobalt are distinguished by their very high hardness. The (CoFe)YB rods in [24] establishes 1336 HV (100 g load, dwell time 10 s) and in [25] CoNiTaB even 1410 HV (load 5 N, 10 s dwell time).

Taking into account that Co is a natural ferromagnetic material with a magnetic moment of 1.7–1.75 μB. Moreover, this element is characterized by spin-orbit coupling stronger than pure iron and it is extremely important during designing materials for practical applications, i.e., small size inductor footprint or transformers [26,27]. CoFeSiB soft magnetic alloys are excellent candidates for such applications due to high electrical resistivity. The most popular soft magnetic Co-based alloy is a material called Vitrovac and its modifications [28,29] which are characterized by relatively low coercivity, high saturation magnetization, low eddy current and high magnetic permeability.

It was observed that the Co-based alloys reduced Barkhausen jumps on the magnetic field in toroidal samples from λs = 30·10^−6^ for Fe_84_B_16_ to λs = 10^−6^ for Co_70_Fe_5_Si_15_B_10_ [30].

Recently, Nosenko and coworkers [31] have shown that selective Cr addition and optimum thermal treatment could decrease saturation induction, which is extremely important in the perspective application of Co-based alloys as supersensitive fluxgate sensors.

Co-based alloys are in the field of interest because of their mechanical and magnetic properties, which are very good, and are also investigated in this work. However, ribbons, rods, or layers are usually investigated, but there is not sufficient information about the properties of powders obtained by gas-atomization. Studying the properties of powder in the initial state and after annealing is important to understand further research on samples produced from powders like BMG performed by additive manufacturing or layers. In this work, CoBFeSiNb and CoBFeTaSiCu powders will be investigated.

## 2. Materials and Methods

In this work, two Co-based alloys were examined, first alloy- Co_47.6_B_21.9_Fe_20.4_Si_5.1_Nb_5_% at, Co_58.6_B_4.9_Fe_23.8_Si_3_._0_Nb_9.7_% wg and second alloy- Co_42_B_26.5_Fe_20_Ta_5.5_Si_5_Cu_1_% at, Co_48.8_B_5.6_Fe_22_Ta_19.6_Si_2.8_Cu_1.3_% wg. Alloys used in this research were obtained by alloying pure chemical elements: Co (99.99%), Fe (99.97%), B (99.9%), Si (99.999%), Nb (99.95%), Cu (99.999%), Ta (99.995%). The precise composition of the alloys is shown in Table 1.

Alloying was performed in an induction vacuum furnace VIM-LAB 50-60. Each alloy was maintained at high temperature for 1 h in the furnace to melt and mix all elements and then cast. After alloying, the cast bars were remelted under vacuum in a crucible, and then the metal was atomized using a jet of high-pressure argon. The powders obtained were sieved using vibrating sieves with a grid of 80, 50 and 20 µm. Then fractions 20–50 µm and 50–80 µm were selected for further investigations. The granulations obtained by sieving were analyzed by liquid particle size analysis (PSA) with the Anton Parr Particle Size Analyzer PSA 1190, Anton Paar GmbH, 8054 Graz, Austria. Obscuration of all measurements was set between 5–15%. Before measurements were made, ultrasound dispersions of the samples were performed. The reconstruction mode was Fraunhofer. Optical and chemical composition analyses of the obtained particles were carried out by Scanning Electron Microscopy (SEM) Tescan Vega with Energy Dispersive Spectroscopy (EDS), module with material and topographic contrast (BSE and SE), TESCAN ORSAY HOLDING, a.s., Brno, Czech Republic. The acceleration voltage of the electrons for analysis was set at 20V. The amorphous state of the alloys and the presence of the crystalline phase were investigated by X-ray diffraction (XRD) using Rigaku Ultima IV Diffractometer, Rigaku Corporation, Tokyo, Japan with Cu Kα irradiation (λ = 1.5406 Å). The range of X-ray scanning was 15–95° 2θ with a step of 0.05°. The electric parameters used for the analysis were 40 kV/40 mV. To establish the onset of the temperature of glass transition (T_g_) and crystallization (T_x_) Differential Thermal Analysis (DTA) was performed on STA 449 F3 Jupiter by NETZSCH factory thermal analyzer, NETZSCH-Gerätebau GmbH, 95100 Selb, Germany. The heating rate for DTA was 40 C/min. The above-described characterization of two granulations of each Co-based alloy leads to focus on 50–80 µm powder granulation for the next step of the experiment. That next step included the annealing of the samples. The 50–80 µm particle size powder was divided into 3 groups. The first group was powder particles in the initial state- as-atomized, the second group was annealed at T_g_ and the third group was annealed at T_x_, respectively, and the temperatures were matched for the first and second alloy. The samples were annealed for 20 min in an atmosphere of inert gas (argon) in a Lt15/12/C450 Nabertherm furnace, Nabertherm GmbH, 28865 Lilienthal, Germany. Characteristic temperatures (T_g_ and T_x_) were taken into account in this research because in those temperatures usually the biggest changes in mechanical parameters occurred. In [32,33] the first biggest change in hardness value of amorphous samples was observed between samples annealed at peak of T_x_ and annealed at T_g_. The first noticeable change in fracture toughness and fracture mechanism of samples were observed between samples as-cast and annealed at T_g_.

The time of annealing across the literature has ranged from 5 min [34] to hours [35]. By experience, and the work of other researchers [36,37], it was decided that 20 min is proper to observe the first changes in properties of the examined samples in this research.

After the samples were annealed and cooled outside the furnace in an inert atmosphere, X-ray diffraction (XRD) and SEM tests were re-performed. Next, the mechanical parameters of these three groups of samples were examined by using nanoindentation. Nanoindentations were taken on an Anton Paar Tester NHT3 on the STeP 4 platform with Berkovich type tip, Anton Paar GmbH, 8054 Graz, Austria, calibrated before measurements on fused silica samples. Measurements were taken at room temperature, the applied load was set at a level of 35 mN, a pause of 30 s was observed and rate of load and unloading was 70 mN/min. The distance between the indentations was at least 1.5x the width of the indent. Calculations of the hardness and the elastic module were performed using the Oliver and Parr method [38]. Indentation hardness (H_IT_) was calculated from Equation (1) as a ratio of the maximum test force (F_max_) to the projected contact area (A_p_)

H_IT_ = F_max_/A_p_, (1)

A_p_ for the Berkovich tip is calculated from Equation (2) as a square of the contact depth of the indent (h_c_) multiplied by 24.5. h_c_ is measured as the maximum depth of the indent (h_max_), while F_max_ is obtained, and reduced by elastic deformation of the surface following Sneddon discovery [39] (3).

A_p_ = 24.5 h_c_^2^, (2)

h_c_ = h_max_ − 0.75 F_max_/S, (3)

S in (3) represents the contact stiffness calculated as a slope of the plot during unloading. To convert the indentation hardness from MPa to Vickers (HV) the (4) equations were used (proper for Berkovich indenter tip).

HV_IT_ ≈ H_IT_/10.80, (4)

Elastic indentation module (E_IT_) was calculated using Equation (5), where: ν_s_, ν_i_- Poisson’s ratio of sample and indenter, respectively, E_i_- reduced elastic module and indenter elastic module, respectively.

E_IT_ = (1 − ν_s_^2^)/[1/E_r_ − (1 − ν_i_^2^)/E_i_], (5)

The values of ν_i_ and E_i_ were taken from the documentation of the indenter delivered by the manufacturer. ν_s_ was estimated by a review of the literature [40,41,42] and established at 0.315.

The static hysteresis loops were measured at room temperature using a LakeShore VSM 7307 vibrating sample magnetometer, Lake Shore Cryotronics, Inc., Ohio 43082, USA, working under an external magnetic field up to 2T. The Mössbauer spectra were collected at room temperature using a Polon Mössbauer spectrometer, Polon, Warsaw, Poland, working in transmission geometry with the 57Co source in the Rh matrix (of the activity of 50 mCi). The studies were carried out on samples crushed to the powder in order to obtain a representation of the entire volume. The analysis was performed using a thin absorber approximation. The Mössbauer spectra were fitted with the WinNORMOS for Igor 6.04 package.

## 3. Results

### 3.1. Particle Size Distribution

**Figure 2:**
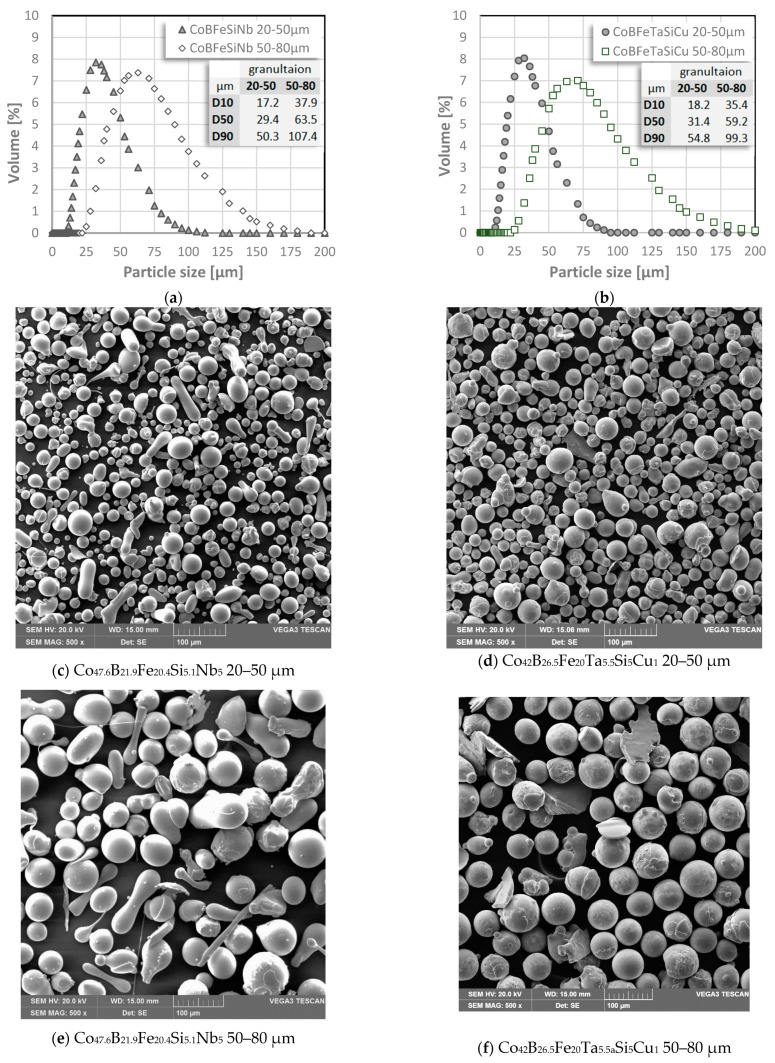
Particle size analysis of (**a**) Co_47.6_B_21.9_Fe_20.4_Si_5.1_Nb_5_ and (**b**) Co42B26.5Fe20.0Ta5.5Si5Cu1% at fraction 20–50 and 50–80 μm. (**c**–**f**) Example of alloys analyzed with SEM SE mag. ×500.

Obtained through the alloying of chemical elements and atomization, Co-based powders of the first and second alloys form regular, spheric-shaped particles. By sieving the first and second alloy, two granulations to test, 20–50 µm and 50–80 µm were received successfully. The results of the particle size analysis showed that 50% of the particle size (D50) for the granulation of 20–50 μm granulation had a diameter less than or equal to 31.41 μm and 29.43 μm, for the first and second alloys, respectively. D50 for 50–80 μm sets on 59.19 μm and 63.50 μm, analogously. The analysis plots and images of the tested samples are shown in Figure 2. Further research was carried out on a higher granulation of alloys- 50–80 µm.

### 3.2. EDS Analyzes/Chemical Composition Homogeneity of Particles

**Figure 3:**
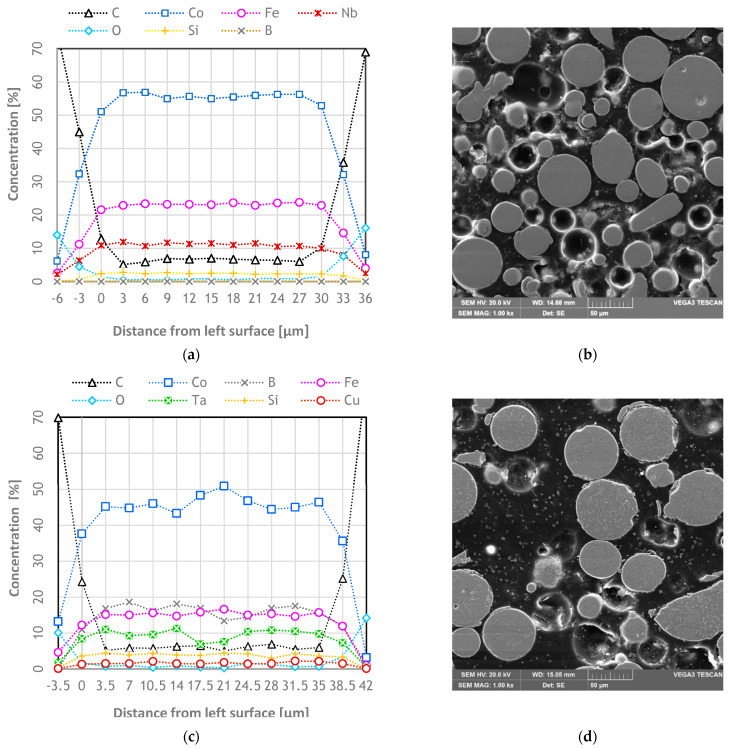
Example of EDS analysis of chemical composition homogeneity and SEM image of analyzed metallography section of (**a**,**b**) Co_47.6_B_21.9_Fe_20.4_Si_5.1_Nb_5_% at. and (**c**,**d**) Co_42_B_26.5_Fe_20.0_Ta_5.5_Si_5_Cu_1_% at.

SEM EDS analysis has shown a steady level of the amount of each chemical element inside the particles of both alloys. For the first alloy, the maximum difference between the maximum and minimum concentrations of each element was 2.5%. For the second alloy, maximum differences were 7.6% for Co and around 5% for Fe and Ta. For the rest of the elements in the second alloy, there was less than a 2.5% difference between the maximum and minimum concentrations. O and C rising on the edges of the graph came from the background of the particles. The described analysis and images of the analyzed metallographic section of the particles are shown in Figure 3.

### 3.3. Differential Thermal Analysis

**Figure 4:**
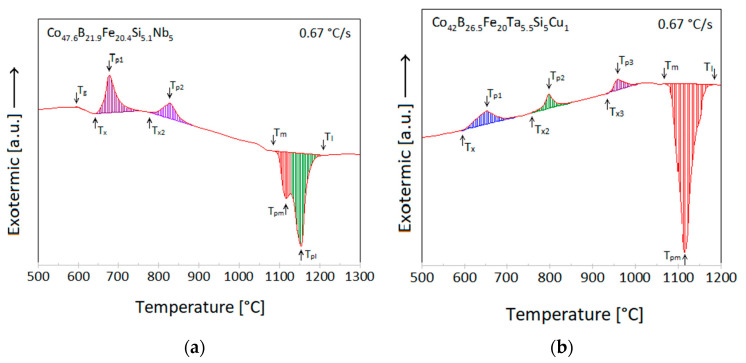
Differential Thermal Analysis (DTA) of two Co-based alloys.

Conducting DTA of the first alloy shows the existence of an exothermal peak corresponding to the temperature of glass transition the T_g_ and set at 598 °C. The next two exothermic peaks were observed, and the first one onset was 656 °C. The last peak with two maxims was endothermic and corresponded to the process of melting (T_m_)- onset at 1074 °C. Analysis by DTA second alloying did not point out Tg, but by studying the literature the T_g_ of the second alloy was established at 545 °C for the next steps of the experiment [43]. However, three exothermic peaks were observed for the second alloy, and the first of them has an onset at 595 °C. The endothermal peak occurred at 1069 °C (T_m_). The DTA plot printed after the analysis is shown in Figure 4. The registered temperatures are shown in Table 2.

### 3.4. Annealing and X-ray Diffraction Test (of As-Atomized and Annealed Samples)

**Figure 5:**
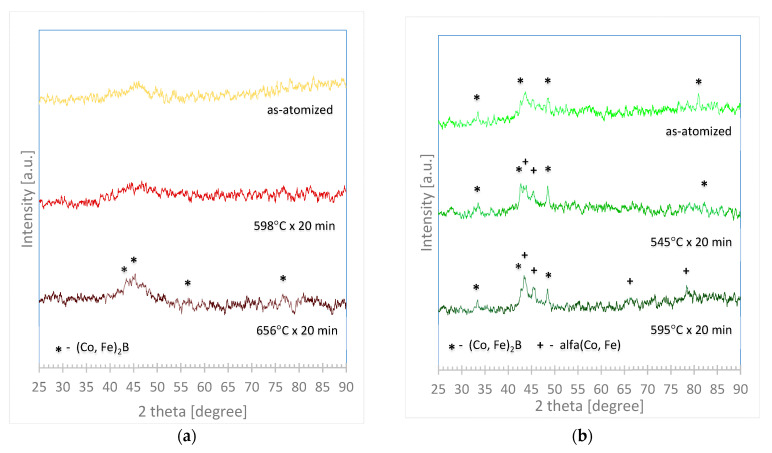
X-ray diffractogram plots of (**a**) Co_47.6_B_21.9_Fe_20.4_Si_5.1_Nb_5_ and (**b**) Co_42_B_26.5_Fe_20_Ta_5.5_Si_5_Cu_1_ powder as atomized, annealed at glass transition temperature (T_g_) and annealed at first crystallization temperature (T_x_).

Annealing of 50–80 µm powders of first and second alloys was carried out. XRD plots are shown in Figure 5. The XRD test of the first alloy in the atomized and annealed state at Tg did not show any diffraction peaks. Annealing at T_x_ caused peaks of diffraction that may be indicated on (Co, Fe)_2_B. Particles of the second alloy in the as-atomized state showed some disturbances of the plot, which suggest that the atoms are more repeatable arranged than an as-atomized sample of the first alloy. The pattern of XRD of the second sample in the as-atomized state corresponds to (Co, Fe)_2_B. High intensity and broad peaks can be observed on XRD plots for the second alloy that was annealed at T_g_ and annealed at T_x_. Compared to the literature, [25,26,27], from analysis of the XRD patterns of diffracted X-rays for the second alloy annealed at T_g_, it can be concluded that these peaks correspond to (Co, Fe)_2_B and some lower intense peaks indicated to α-(Co, Fe). The last graph of the second alloy, for the powder annealed at T_x_, had an instance and broad peaks indicating the presence of crystalline peaks of both (Co, Fe)_2_B and α-(Co, Fe).

### 3.5. SEM Analysis

**Figure 6:**
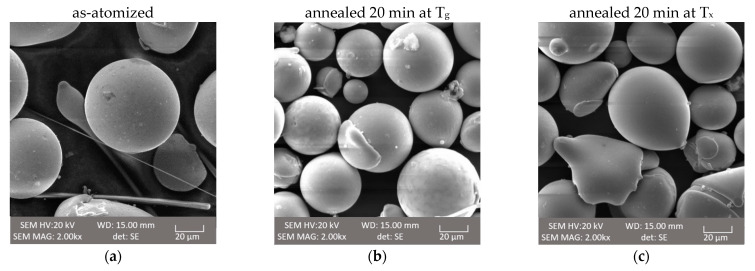

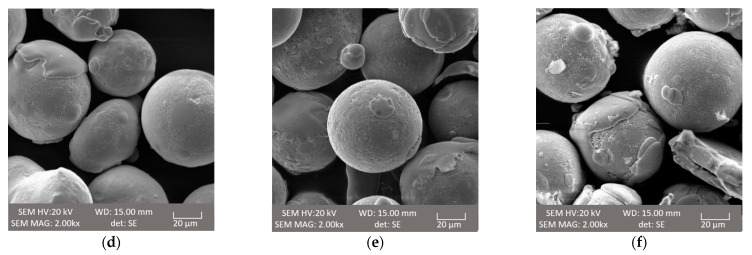
SEM image, topography contrast of (**a**–**c**) Co_47.6_B_21.9_Fe_20.4_Si_5.1_Nb_5_, (**d**,**e**) Co_42_B_26.5_Fe_20_Ta_5.5_Si_5_Cu_1_; (**a**,**d**) powders as atomized, (**b**,**e**) annealed at glass transition temperature (T_g_), (**c**,**f**) annealed at first crystallization temperature (T_x_).

Analyzed SEM samples of the as-atomized powder of the first alloy present regular spheric particles with some splats on the surfaces. However, no splats detached from the spheric particles were observed. The surface of the particles and splats were plain without any irregularities. The microscopic observation did not show any changes on the surface of the particles that occurred by annealing of the powder samples, neither at T_g_ nor at T_x_. The second alloy as-atomized powder SEM analysis also exhibits a regular spheric shape of the particles and presents some splats joined to the spheric particles. The surface of the particles was irregular. Like before, annealing at T_g_ and T_x_ of the second alloy did not cause visible changes on the particles. Representative samples of each powder are shown in Figure 6.

### 3.6. Nanoindetation

**Figure 7:**
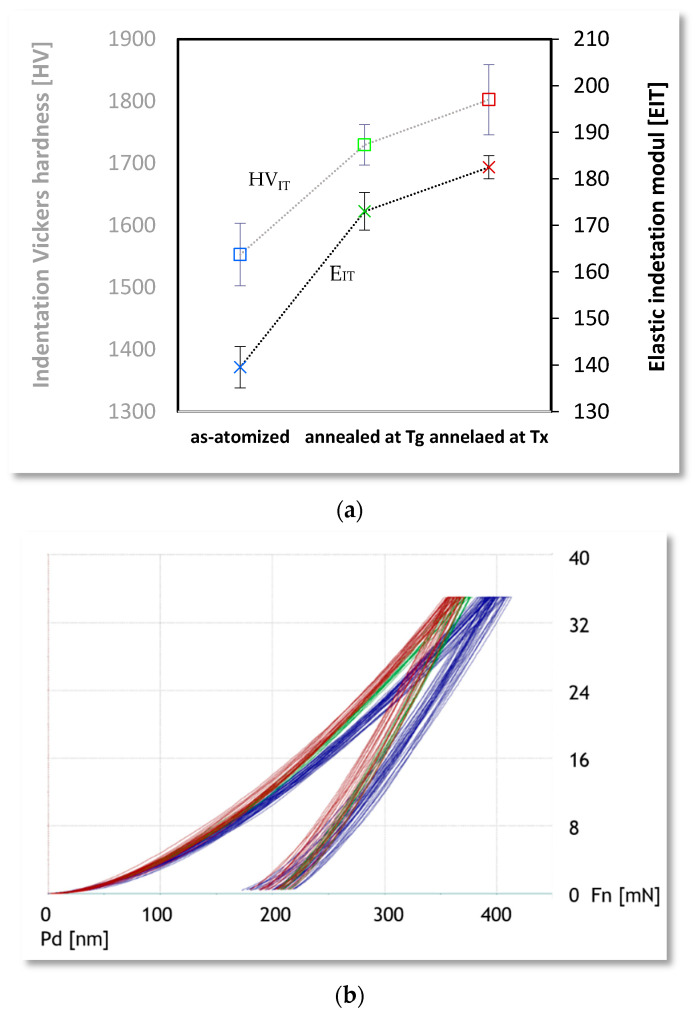
(**a**) Plots of the nanoindentation test, force to the depth of penetration (blue—as-atomized; green—annealed at T_g_, glass transition temperature; brown- annealed at T_x_, crystallization temperature), (**b**) graphic presentation of nanoindentation test results.

The mean hardness (H_IT_) value of the second alloy calculated from nanoindentation tests for atomized particles was 16,768.64 MPa (or 1552.96 HV), for annealed at T_g_ 18,562.29 MPa (1721.85 HV) and T_x_ 19,463.81 MPa (1221.52 HV). These results indicate that annealing in this experiment causes an increase in the mean hardness for annealing of 76.31 MPa for annealing at T_g_ and 149 MPa for annealing at T_x_. Likewise, the elastic indentation module increases after annealing as-atomized powder. E_IT_ set on 139.54 GPa, 173.85 GPa and 182.5 GPa respectively, for the as-atomized, annealed at T_g_ and annealed at T_x_. The results described are shown in Table 3. Examples of plots obtained from nanoindentation and graphic results are shown in Figure 7.

### 3.7. Mössbauer Studies

**Figure 8:**
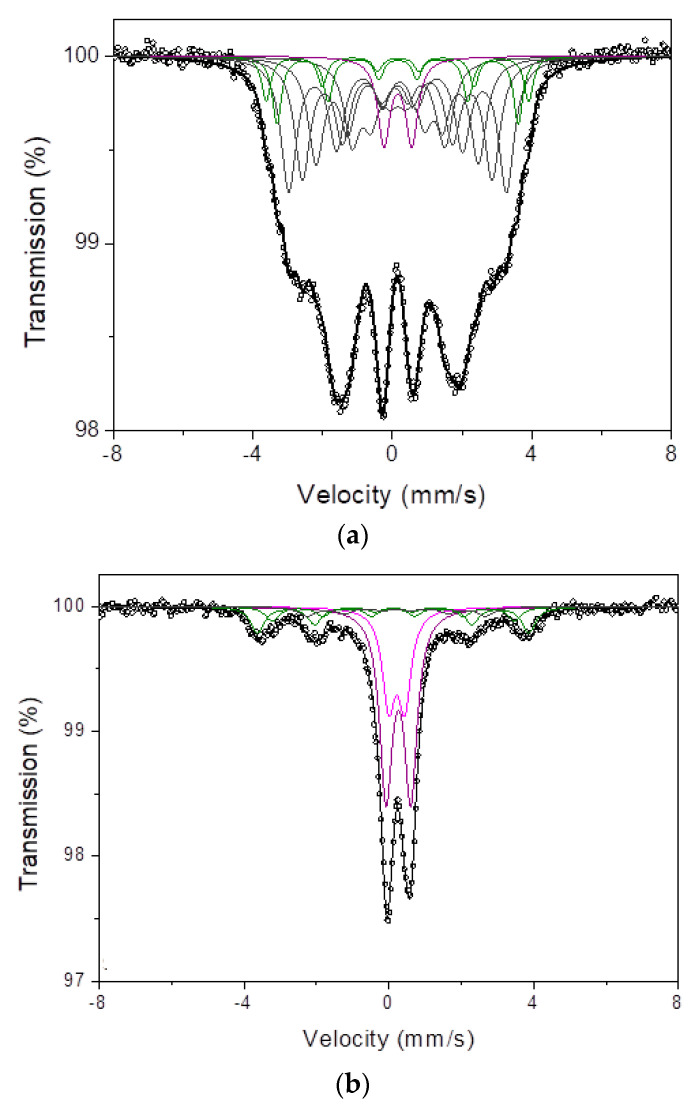
Analyzed Mössbauer spectrum of the as-atomized (**a**) Co_47.6_B_21.9_ Fe_20.4_Si_5.1_Nb_5_ and (**b**) Co_42_B_26.5_Fe_20_Ta_5.5_Si_5_Cu_1_.

As is well known, the Mössbauer spectroscopy is a very sensitive method for the investigation of the structure and magnetic state of materials. X-ray diffraction revealed an amorphous structure; however, in the case of Mössbauer spectroscopy, some phases were detected. This difference is due to the sensitivities of each method. The measured spectrum of Co_47.6_Fe_20.4_B_21.9_Si_5.1_Nb_5_ was deconvoluted in a doublet corresponding to the disordered Fe-based phase and in sextets that are related to Fe_2_B and Fe-Nb-B phases. Taking into account analysis of annealed samples, a change in phase constitution was not observed; however, successive increase of Fe_2_B phase at the expense of two other phases was detected. All parameters, such as, isomer shift IS, quadrupole splitting QS and hyperfine field induction Bhf for all studied samples were collected in Table 4. For the Co_42_Cu_1_Fe_20_Ta_5.5_B_26.5_Si_5_ alloy samples, the Mössbauer spectra collected at room temperature were deconvoluted in three phases: Fe-Cu corresponding to paramagnetic doublet, Fe_2_B, and Fe-B-Nb corresponding to ferromagnetic sextets. The analysis of spectra of annealed samples expansion of Fe_2_B phase at the expense of other two phases. Table 4 contains all parameters of the Mössbauer spectra. The room temperature Mössbauer spectrums of the as-atomized first and second alloy samples are shown in Figure 8.

### 3.8. Magnetic Properties

**Figure 9:**
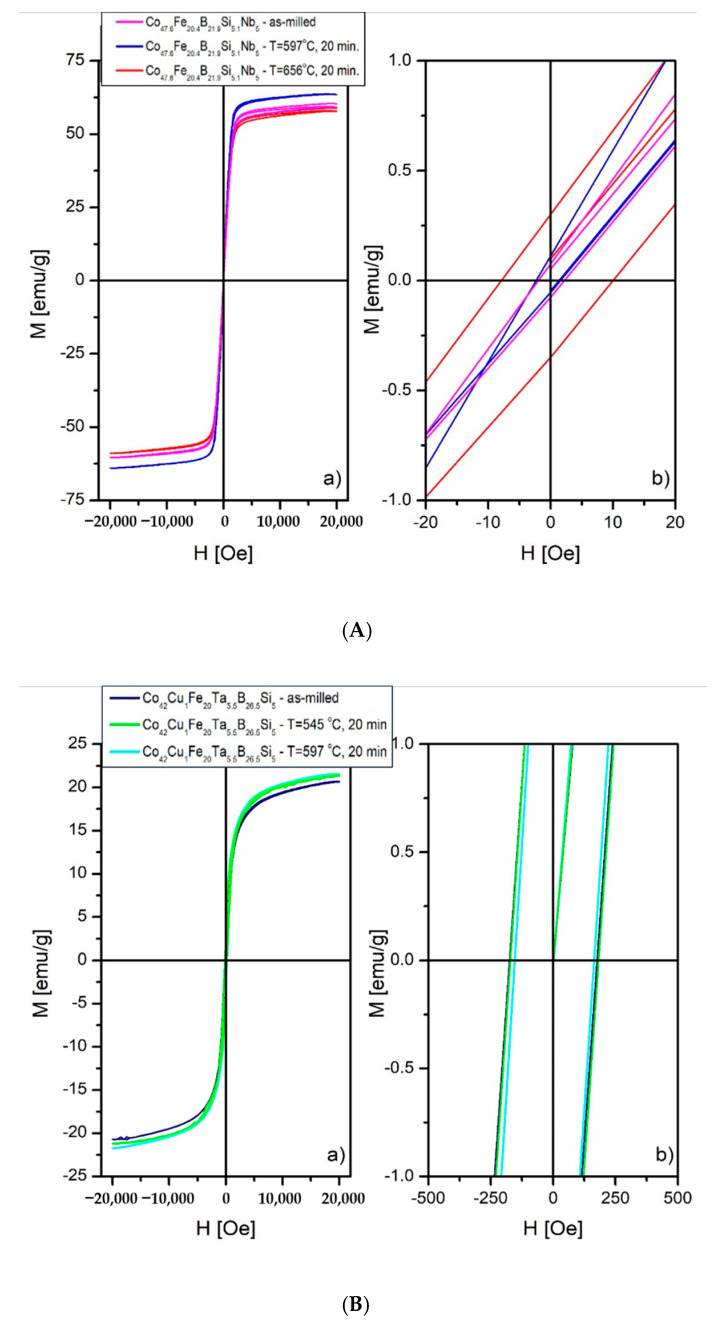
The room temperature hysteresis loops of the (**A**) Co_47.6_B_21.9_Fe_20.4_Si_5.1_Nb_5_, (**B**) Co_42_B_26.5_Fe_20_Ta_5.5_Si_5_Cu_1_ alloy; (as-atomized, and after heat treatment).

In order to reveal magnetic properties and classify the powder produced into some group of magnetic materials, the static hysteresis loops at room temperature were measured (Figure 9). In the case of samples of the Co_47.6_B_21.9_Fe_20.4_Si_5.1_Nb_5_ alloy, the saturation magnetization is 59.4, 63.6 and 58.1 Am^2^/kg for as-atomized, annealed at 597 °C and 656 °C, respectively. The phase contained Nb and Ta were detected by Mössbauer spectroscopy due to its higher sensitivity compared to the XRD diffraction. The coercivity equalled 319, 321 and 690 A/m. The saturation magnetization for Co_42_B_26.5_Fe_20_Ta_5.5_Si_5_Cu_1_ alloy sets on the level of 20.6, 21.4 and 21.6 Am^2^/kg for the as-atomized sample, annealed at a temperature of 597 °C and 656 °C, respectively. Analysis of the hysteresis loops allowed revealing values of coercivity, which were 13.9, 12.7 and 13.9 kA/m for the as-atomized sample, annealed at temperature 597 °C and 656 °C, respectively.

## 4. Discussion

Based on the results shown in this work, atomization was confirmed as a suitable method to obtain powder-form alloys with high glass formation ability (GFA), including Co-based amorphous alloys such as Co_47.6_B_21.9_Fe_20.4_Si_5.1_Nb_5_ % at. and Co_42_B_26.5_Fe_20_Ta_5.5_Si_5_Cu_1_% at. Other research also shows the proven accuracy of the method in developing regular spheric powder particles with the bell-shaped plot of the particle size distribution [44,45,46]. 

EDS analysis showed that the chemical composition of the first alloy, namely Co_47.6_Fe_20.4_B_21.9_Si_5.1_Nb_5.0_% at, was suitable to gain a homogeneous practice through the atomization process. All particles, from the fraction 20–50 µm and 50–80 µm, had a steady level of concentration of each chemical composition. This was also confirmed by a microscopic observation shown in Figure 2b. However, the second alloy had less homogeneity. Some fluctuations of the concentration elements were observed, suggesting that the atomization process of Co_42_Cu_1_Fe_20_Ta_5.5_B_26.5_Si_5_ should be improved. These results are in line with [47] which confirm the homogeneous chemical composition obtained by atomization as in the first alloy.

Differential thermal analysis for the first alloy in the form of powder, granulation 20–50 µm, exhibited for the T_g_ peak 620 °C and the T_x_ inflexion 663 °C. According to the work [48,49] for the same chemical composition of the alloy as the first alloy, but in rod form, the T_g_ peak is set at 580 °C and the inflection of T_x_ 623 or 635 °C for [48,49], respectively. Since the heat rate in the experiments in this work and in [48,49] was the same (40 K/min), it could be concluded that the form of the material tested causes translation to the right graph of DTA for about 40 °C.

For the second alloy in the form of powder 20–50 µm, DTA in this work did not show a T_g_ peak while according to [43] for the alloy identical to second but in rods T_g_ set at 623 °C. The T_x_ onset read from DTA in this work was set at 595 °C and is 82 °C lower than the temperature measured in [43]. The difference between temperatures might be due to the different forms of the tested samples and the different heat rates. In [43] the heat rate was twice lower (20 K/min) than in this work.

The results of the first and second XRD pattern confirm those of earlier studies, such as [43,50,51,52]. Where respectively, the first two works confirm that alloys with similar elements developed first a crystal phase (Co, Fe) 2B and [50,51,52] shows a crystallization of α-(Co, Fe) which was developed only in the second alloy after annealing. The first alloy after atomization was fully amorphous, while the second already had some crystal phase (in an as-atomized state). In the study literature, the first alloy has a ΔT_x_ = 52 or 44 °C [48,49], and the second ΔT_x_ = 42 °C [43], which may suggest that the first alloy has a better glass-forming ability that can explain the different states of particles in the samples as atomized.

After annealing, the hardness of the powder particles increases-for annealing in T_x_ by about 11% and for annealing in T_x_ for 26%. The same as the elastic module, for T_g_ annealing increased by 24% and for T_x_ by 31%. All of these increases are caused by increasing the amount of crystal phase in samples and correspond to the results of other researchers [53,54]. However, other research refers to Fe-based alloys because those based on Co have not been studied well enough. Increasing hardness is explained as the first state being a relaxation of the structure and the second as a growing crystal hard phase. In the research [54] there is also information on the short range of decreasing hardness that is the cause of initial crystallization without the possibilities of relaxation, but in this work that case did not appear. The same studies explain and confirm the increase of the elastic module through the annealing of amorphous samples.

The Mössbauer spectroscopy studies revealed traces of some phases, such as the paramagnetic disordered Fe-based phase and ferromagnetic Fe_2_B and Fe-Nb-B phases for the Co_47.6_B_21.9_Fe_20.4_Si_5.1_Nb_5_alloy. The content of each phase was changed slightly during heat treatment. Similar behavior was detected in the Co_42_B_26.5_Fe_20_Ta_5.5_Si_5_Cu_1_ alloy samples. The spectra contain traces of the paramagnetic Fe-Cu phase and the ferromagnetic Fe_2_B and Fe-Nb-B phases. Magnetic studies revealed that in the case of the Co_47.6_B_21.9_Fe_20.4_Si_5.1_Nb_5_alloy, it is a soft magnetic material both: in the as-atomized state and after heat treatment. Co_47.6_Fe_20.4_B_21.9_Si_5.1_Nb_5_ alloy establishes the saturation magnetization 59.4, 63.6 and 58.1 Am^2^/kg for as-atomized, annealed at T_g_ and T_x_, respectively. Taking into account the results provided by Ackland et al. [28] the measured values are almost twice lower. It is probably caused by the degradation of a structure during atomization. In the case of saturation magnetization, no strong deviations were observed, which suggests that the material is structurally stable in the as-atomization state and after annealing. Results for the coercivity equalled 319, 321 and 690 A/m, respectively, for the same states as above. The results correspond well to the data provided by other authors [28,55,56,57,58].

Significantly different properties were measured for samples of the Co_42_Cu_1_Fe_20_Ta_5.5_B_26.5_Si_5_ alloy. The saturation magnetization was almost three times lower than for the first alloy, namely 20.6, 21.4 and 21.6 Am^2^/kg for the as-atomized sample, annealed at T_g_ and T_x_, respectively. Compared to the data provided by Ackland et al. [28] and Mohapatra et al. [58] our results are lower or comparable, which is induced by a lower Fe content and other preparation conditions. Coercivity for the second alloy was even 10 times lower than for a first alloy 13.9, 12.7 and 13.9 kA/m for the as-atomized sample, annealed at T_g_ and T_x_, respectively. Such values qualify the produced samples as semi-hard magnetic materials. Moreover, similar to the first alloy, the fluctuations of saturation magnetization were not observed. As is visible in Figure 9a, the Nb-doped samples have an almost rectangular shape of hysteresis loops. The rectangular shape of hysteresis loops is typical for axial anisotropy. Most likely, some axial anisotropy is induced by Nb atoms. In the case of samples doped by Ta, the inclination of hysteresis loops is visible in Figure 9b, which means that Ta atoms do not induce an axial anisotropy compared to Nb atoms. In the case of Ta-doped samples, we can talk about circumferential anisotropy. A similar effect was observed in Co-based microwires induced by thermal treatment [59,60].

## 5. Conclusions

▪ Co_47.6_B_21.9_Fe_20.4_Si_5.1_Nb_5_% at. and Co_42_B_26.5_Fe_20_Ta_5.5_Si_5_Cu_1_% at. are suitable alloys to produce powder by atomization and the obtained particles have a chemical homogeneity concentration. However, the second alloy had a slightly smaller homogeneity than the first alloy.
▪ The atomized powder 50–80 µm of Co_47.6_Fe_20.4_B_21.9_Si_5.1_Nb_5_ alloy exhibits a T_g_ = 620 °C and T_x_ = 663 °C on a DTA research. Co_42_Cu_1_Fe_20_Ta_5.5_B_26.5_Si_5_ exhibits the smaller T_x_ (then first alloy), namely 595 °C and does not show any T_g_ sights. Those temperatures are lower than the temperature taken from the literature, which can be caused by a different form of tested samples (powders vs. rods).
▪ The surface of the first and second alloy did not visibly change after annealing at T_g_ nor T_x_.
▪ The annealing for 20 min at T_g_ and the annealing at the T_x_ powder samples of Co_47.6_B_21.9_Fe_20.4_Si_5.1_Nb_5_and Co_42_B_26.5_Fe_20_Ta_5.5_Si_5_Cu_1_ causes an increase of the hardness of the indentation and increase of the elastic indentation module.
▪ Magnetic research shows that the Co_47.6_B_21.9_Fe_20.4_Si_5.1_Nb_5_alloy is a soft magnetic material, both in the atomized state and after annealing state. Co_42_B_26.5_Fe_20_Ta_5.5_Si_5_Cu_1_ shows semi-hard magnetic material properties in both states.

## Figures and Tables

**Table 1:** Chemical composition of the alloys in this work and prunes of the elements used for alloying.

	Elements						
First Alloy	Cobalt	Iron	Boron	Silicon	Niobium	Copper	Tantalum
% at.	47.60	20.40	21.90	5.10	5.00	0.00	0.00
% wg.	58.58	23.79	4.94	2.99	9.70	0.00	0.00
**Second Alloy**	-	-	-	-	-	-	-
% at.	42.00	20.00	26.50	5.00	0.00	1.00	5.50
% wg.	48.75	22.00	5.64	2.77	0.00	1.25	19.60
% at.	99.99	99.97	99.9	99.999	99.95	99.999	99.995

**Table 2:** Characteristic temperature of Co_47.6_B_21.9_Fe_20.4_Si_5.1_Nb_5_ and Co_42_B_26.5_Fe_20_Ta_5.5_Si_5_Cu_1_.

Temperature [°C]	T_g_	T_x_	T_x2_	T_x3_	ΔT_x_	T_m_	T_l_
Co_47.6_B_21.9_Fe_20.4_Si_5.1_Nb_5_	598	656	782	-	58	1074	1144
Co_42_B_26.5_Fe_20.0_Ta_5.5_Si_5_Cu_1_	-	595	768	928	-	1069	1116

**Table 3:** Results of the second alloy nanoindentation test in the as-atomized state, annealed at T_g_ and T_x_.

	As-Atomized			Annealed at T_g_			Annealed at T_x_		
Parameter	H_IT_	HV_IT_	E_IT_	H_IT_	HV_IT_	E_IT_	H_IT_	HV_IT_	E_IT_
Unit	(MPa)	(HV)	(GPa)	(MPa)	(HV)	(GPa)	(MPa)	(HV)	(GPa)
**Mean**	16,768.6	1553.0	139.5	18,592.3	1721.9	173.0	19,463.8	1802.6	182.5
**Standard deviation**	1085.4	100.5	8.9	703.6	65.2	8.1	1221.5	113.1	5.0
**Coefficient of variation [%]**	6.5	6.5	6.4	3.8	3.8	4.7	6.3	6.3	2.7

H_IT_—indentation hardness, HV_IT_—Vickers hardness calculated from indentation hardness HV_IT_ = H_IT_/10.80, E_IT_—indentation elastic module.

**Table 4:** The parameters of the Mossbauer spectra of studied samples. D-doublet, S-Sextet.

Component	IS (mm/s)	QS (mm/s)	B (T)	FWHM (mm/s)	A (%)	Compound
**Co_47.6_B_21.9_Fe_20.4_Si_5.1_Nb_5_ as-atomized**						
D1	0.16	0.79	-	0.42	7	Fe-based
S1	0.16	−0.02	23.4	0.32	5	Fe_2_B
S2	0.16	−0.01	21.5	0.32	8	
S3	0.18	−0.06	19.4	0.54	23	Fe-B-Nb
S4	0.15	−0.01	16.9	0.54	20	
S5	0.11	0.07	14.5	0.54	17	
S6	0.10	0.01	11.3	0.54	14	
S7	0.17	0.03	8.3	0.54	6	
**Co_47.6_B_21.9_Fe_20.4_Si_5.1_Nb_5_ annealed at T = 597 °C for 20 min.**						
D1	0.19	0.76	-	0.36	5	Fe-based
S1	0.15	−0.02	23.5	0.32	7	Fe_2_B
S2	0.15	−0.01	21.6	0.32	9	
S3	0.16	−0.06	19.7	0.52	20	Fe-B-Nb
S4	0.15	−0.02	17.3	0.52	20	
S5	0.10	0.07	14.7	0.52	16	
S6	0.07	0.04	11.2	0.52	12	
S7	0.17	0.05	8.1	0.52	11	
**Co_47.6_B_21.9_Fe_20.4_Si_5.1_Nb_5_ annealed at T = 656 °C for 20 min.**						
D1	0.15	0.78	-	0.41	4	Fe-based
S1	0.17	−0.02	23.3	0.32	9	Fe_2_B
S2	0.16	−0.01	21.5	0.32	9	
S3	0.19	−0.06	19.5	0.55	22	Fe-B-Nb
S4	0.15	−0.01	16.8	0.55	20	
S5	0.11	0.07	14.5	0.55	15	
S6	0.09	0.01	11.3	0.55	15	
S7	0.17	0.03	8.3	0.55	6	
**Co_42_ B_26.5_Fe_20_Ta_5.5_Si_5_Cu_1_ as- atomized**						
D1	0.22	0.44	-	0.43	24	Fe-Cu
D2	0.27	0.68	-	0.43	47	
S1	0.12	0.00	23.8	0.45	14	Fe_2_B
S2	0.13	0.00	21.7	0.45	8	
S3	0.21	0.00	19.1	0.59	7	Fe-B-Nb
**Co_42_ B_26.5_Fe_20_Ta_5.5_Si_5_Cu_1_annealed at T = 545 °C for 20 min.**						
D1	0.21	0.41	-	0.41	18	Fe-Cu
D2	0.28	0.66	-	0.41	51	
S1	0.11	0.00	23.5	0.52	12	Fe_2_B
S2	0.09	0.00	21.5	0.52	12	
S3	0.21	0.00	13.9	0.52	6	Fe-B-Nb
**Co_42_ B_26.5_Fe_20_Ta_5.5_Si_5_Cu_1_annealed at T = 597 °C for 20 min.**						
D1	0.24	0.46	-	0.41	26	Fe-Cu
D2	0.28	0.68	-	0.41	40	
S1	0.11	0.00	23.8	0.39	10	Fe_2_B
S2	0.12	0.00	21.7	0.39	11	
S3	0.08	0.00	19.1	0.39	5	Fe-B-Nb
S4	0.20	0.00	13.7	0.58	8	

## Data Availability

Data is contained within the article.

## References

[1] Pilarczyk W. (2017). Struktura i Właściwości Masywnych Szkieł Metalicznych w Stanie Po Wytworzeniu i Po Procesie Spawania Laserowego.

[2] Stoica M. (2017). Fe-Based Bulk Metallic Glasses: Understanding the Influence of Impurities on Glass Formation.

[3] Żrodowski Ł., Wróblewski R., Choma T., Morończyk B., Ostrysz M., Leonowicz M., Łacisz W., Błyskun P., Wróbel J.S., Cieślak G. (2021). Novel Cold Crucible Ultrasonic Atomization Powder Production Method for 3D Printing. Materials.

[4] Liu X., Bi J., Meng Z., Li R., Li Y., Zhang T. (2021). Tribological Behaviors of High-Hardness Co-Based Amorphous Coatings Fabricated by Laser Cladding. Tribol. Int..

[5] Lin T.-J., Sheu H.-H., Lee C.-Y., Lee H.-B. (2021). The Study of Mechanical Properties and Corrosion Behavior of the Fe-Based Amorphous Alloy Coatings Using High Velocity Oxygen Fuel Spraying. J. Alloy Compd..

[6] Ciftci N., Yodoshi N., Armstrong S., Mädler L., Uhlenwinkel V. (2020). Processing Soft Ferromagnetic Metallic Glasses: On Novel Cooling Strategies in Gas Atomization, Hydrogen Enhancement, and Consolidation. J. Mater. Sci. Technol..

[7] Zhao Y.F., Si J.J., Song J.G., Yang Q., Hui X.D. (2014). Synthesis of Mg–Zn–Ca Metallic Glasses by Gas-Atomization and Their Excellent Capability in Degrading Azo Dyes. Mater. Sci. Eng. B.

[8] Shi Y., Lu W., Sun W., Zhang S., Yang B., Wang J. (2022). Impact of Gas Pressure on Particle Feature in Fe-Based Amorphous Alloy Powders via Gas Atomization: Simulation and Experiment. J. Mater. Sci. Technol..

[9] Katz-Demyanetz A., Koptyug A., Popov V.V. In-Situ Alloying as a Novel Methodology in Additive Manufacturing. Proceedings of the 2020 IEEE 10th International Conference on Nanomaterials: Applications Properties (NAP).

[10] Deng L., Wang S., Wang P., Kühn U., Pauly S. (2018). Selective Laser Melting of a Ti-Based Bulk Metallic Glass. Mater. Lett..

[11] Nong X.D., Zhou X.L., Ren Y.X. (2019). Fabrication and Characterization of Fe-Based Metallic Glasses by Selective Laser Melting. Opt. Laser Technol..

[12] Popov V.V., Grilli M.L., Koptyug A., Jaworska L., Katz-Demyanetz A., Klobčar D., Balos S., Postolnyi B.O., Goel S. (2021). Powder Bed Fusion Additive Manufacturing Using Critical Raw Materials: A Review. Materials.

[13] Ding P., Mao A., Zhang X., Jin X., Wang B., Liu M., Gu X. (2017). Preparation, Characterization and Properties of Multicomponent AlCoCrFeNi_2.1_ Powder by Gas Atomization Method. J. Alloy Compd..

[14] Yang T., Cai B., Shi Y., Wang M., Zhang G. (2021). Preparation of Nanostructured CoCrFeMnNi High Entropy Alloy by Hot Pressing Sintering Gas Atomized Powders. Micron.

[15] Han C., Fang Q., Shi Y., Tor S.B., Chua C.K., Zhou K. (2020). Recent Advances on High-Entropy Alloys for 3D Printing. Adv. Mater..

[16] Sumita M., Hanawa T., Ohnishi I., Yoneyama T., Milne I., Ritchie R.O., Karihaloo B. (2003). 9.04—Failure Processes in Biometallic Materials. Comprehensive Structural Integrity.

[17] Klement W., Willens R.H., Duwez P. (1960). Non-Crystalline Structure in Solidified Gold–Silicon Alloys. Nature.

[18] Msetra Z., Khitouni N., Suñol J.J., Khitouni M., Chemingui M. (2021). Characterization and Thermal Analysis of New Amorphous Co_60_Fe_18_Ta_8_B_14_alloy Produced by Mechanical Alloying. Mater. Lett..

[19] Liu C., Li Q., Huo J., Yang W., Chang L., Chang C., Sun Y. (2018). Near Room-Temperature Magnetocaloric Effect of Co-Based Bulk Metallic Glass. J. Magn. Magn. Mater..

[20] Wang Q., Zhou J., Zeng Q., Zhang G., Yin K., Liang T., Yang W., Stoica M., Sun L., Shen B. (2020). Ductile Co-Based Bulk Metallic Glass with Superhigh Strength and Excellent Soft Magnetic Properties Induced by Modulation of Structural Heterogeneity. Materialia.

[21] Kim J.T., Hong S.H., Kim Y.S., Park H.J., Maity T., Chawake N.M., Prashanth K.G., Park J.M., Song K.K., Wang W.M. (2019). Co-Cr-Mo-C-B Metallic Glasses with Wide Supercooled Liquid Region Obtained by Systematic Adjustment of the Metalloid Ratio. J. Non-Cryst. Solids.

[22] Dastani M.M., AL-Ali M.H., Moradi M. (2019). Influence of Current Annealing on the Magneto-Impedance Response of Co-Based Ribbons Arising from Surface Structural Improvement. J. Non-Cryst. Solids.

[23] Zhao C., Pan L., Li X., Ma L., Liu Q., Wang J. (2018). Optimization of Magnetoimpedance Effect in Co-Based Ribbon by Laser Patterning for Sensor Arrays Application. J. Phys. D Appl. Phys..

[24] Liang X., Li Y., Bao F., Zhu Z., Zhang H., Zhang W. (2021). Roles of Y and Fe Contents on Glass-Forming Ability, Thermal Stability, and Magnetic Properties of Co-Based Co–Fe–Y–B Bulk Metallic Glasses. Intermetallics.

[25] Zhang G., Zhang H., Yue S., Cheng R., Wang A., He A., Dong Y., Ni H., Liu C.-T. (2019). Preparation of Non-Magnetic and Ductile Co-Based Bulk Metallic Glasses with High GFA and Hardness. Intermetallics.

[26] Murray C., Sun S., Gaschler W., Doyle H., Betley T., Kagan C. (2001). Colloidal Synthesis of Nanocrystals and Nanocrystal Superlattices. IBM J. Res. Dev..

[27] Yang H.T., Su Y.K., Shen C.M., Yang T.Z., Gao H.J. (2004). Synthesis and Magnetic Properties of ε-Cobalt Nanoparticles. Surf. Interface Anal..

[28] Ackland K., Masood A., Kulkarni S., Stamenov P. (2018). Ultra-Soft Magnetic Co-Fe-B-Si-Nb Amorphous Alloys for High Frequency Power Applications. AIP Adv..

[29] Pinzón-Escobar E., Montiel H., García A., Alvarez G. (2021). Magnetic and Electrical Properties of Vitrovac/Au/Vitrovac Multilayered Obtained by Means of Magnetron Sputtering. J. Phys. Conf. Ser..

[30] Kekalo I., Stolyarov V., Taranichev V. (1983). The Effect of Annealing on the Laws of Magnetization and Magnetization Reversal in Amorphous Alloys.

[31] Nosenko A.V., Kyrylchuk V.V., Semen’ko M.P., Nowicki M., Marusenkov A., Mika T.M., Semyrga O.M., Zelinska G.M., Nosenko V.K. (2020). Soft Magnetic Cobalt Based Amorphous Alloys with Low Saturation Induction. J. Magn. Magn. Mater..

[32] Rezaei-Shahreza P., Seifoddini A., Hasani S. (2018). Microstructural and Phase Evolutions: Their Dependent Mechanical and Magnetic Properties in a Fe-Based Amorphous Alloy during Annealing Process. J. Alloy Compd..

[33] Hasani S., Rezaei-Shahreza P., Seifoddini A. (2019). Effect of Cu Presence on Evolution of Mechanical and Magnetic Properties in a Novel Fe-Based Bulk Metallic Glass during Partial Annealing Process. Met. Mater. Trans. A.

[34] Wang Q., Chen M., Shao L., Ge Y., Lin P., Chu C., Shen B. (2019). Effects of Structural Relaxation on the Dye Degradation Ability of FePC Amorphous Alloys. J. Non-Cryst. Solids.

[35] Wang Q.-Y., Xi Y.-C., Zhao Y.-H., Liu S., Bai S.-L., Liu Z.-D. (2017). Effects of Laser Re-Melting and Annealing on Microstructure, Mechanical Property and Corrosion Resistance of Fe-Based Amorphous/Crystalline Composite Coating. Mater. Charact..

[36] Xie C., Yang Y., Zhong S., Li S., Deng S. (2017). Formation, Magnetic Properties and Bending Deformation of Fe-Based Amorphous Alloy without Metalloids. J. Alloy Compd..

[37] Onodera R., Kimura S., Watanabe K., Yokoyama Y., Makino A., Koyama K. (2015). Nucleation Control for Fine Nano Crystallization of Fe-Based Amorphous Alloy by High-Magnetic-Field Annealing. J. Alloy Compd..

[38] Oliver W.C., Pharr G.M. (1992). An Improved Technique for Determining Hardness and Elastic Modulus Using Load and Displacement Sensing Indentation Experiments. J. Mater. Res..

[39] Sneddon I.N. (1965). The Relation between Load and Penetration in the Axisymmetric Boussinesq Problem for a Punch of Arbitrary Profile. Int. J. Eng. Sci..

[40] Zhang T., Yang Q., Ji Y., Li R., Pang S., Wang J., Xu T. (2011). Centimeter-Scale-Diameter Co-Based Bulk Metallic Glasses with Fracture Strength Exceeding 5000 MPa. Chin. Sci. Bull..

[41] Wang J., Wang L., Guan S., Zhu S., Li R., Zhang T. (2014). Effects of Boron Content on the Glass-Forming Ability and Mechanical Properties of Co–B–Ta Glassy Alloys. J. Alloy Compd..

[42] Lai L., He R., Ding K., Liu T., Liu R., Chen Y., Guo S. (2019). Ternary Co-Mo-B Bulk Metallic Glasses with Ultrahigh Strength and Good Ductility. J. Non-Cryst. Solids.

[43] Yazici Z., Hitit A., Yalcin Y., Ozgul M. (2016). Effects of Minor Cu and Si Additions on Glass Forming Ability and Mechanical Properties of Co-Fe-Ta-B Bulk Metallic Glass. Met. Mater. Int..

[44] Dai T., Wang N. (2019). Study on Magnetic Properties and Degradability of Gas Atomization Fe-Based (Fe-Si-B-P) Amorphous Powder. J. Supercond. Nov. Magn..

[45] Alvarez K.L., Manuel Martin J., Ipatov M., Dominguez L., Gonzalez J. (2018). Magnetic Properties of Annealed Amorphous Fe_72.5_Si_12.5_B_15_ Alloy Obtained by Gas Atomization Technique. IEEE Trans. Magn..

[46] Ciftci N., Ellendt N., von Bargen R., Henein H., Maedler L., Uhlenwinkel V. (2014). Atomization and Characterization of a Glass Forming Alloy {(Fe_0.6_Co_0.4_)_0.75_B_0.2_Si_0.05_}_96_Nb_4_. J. Non-Cryst. Solids.

[47] Miura A., Dong W., Fukue M., Yodoshi N., Takagi K., Kawasaki A. (2011). Preparation of Fe-Based Monodisperse Spherical Particles with Fully Glassy Phase. J. Alloy Compd..

[48] Zhang G., Wang Q., Yuan C., Yang W., Zhou J., Xue L., Hu F., Sun B., Shen B. (2018). Effects of Cu Additions on Mechanical and Soft-Magnetic Properties of CoFeBSiNb Bulk Metallic Glasses. J. Alloy Compd..

[49] Dong Y., Wang A., Man Q., Shen B. (2012). (Co_1−x_Fe_x_)_68_B_21.9_Si_5.1_Nb_5_ Bulk Glassy Alloys with High Glass-Forming Ability, Excellent Soft-Magnetic Properties and Superhigh Fracture Strength. Intermetallics.

[50] Li L., Sun H., Fang Y., Zheng J. (2016). Co-Based Soft Magnetic Bulk Glassy Alloys Optimized for Glass-Forming Ability and Plasticity. Bull. Mater. Sci..

[51] Shen B., Chang C., Kubota T., Inoue A. (2006). Superhigh Strength and Excellent Soft-Magnetic Properties of [(Co_1−x_Fex)_0.75_B_0.2_Si_0.05_]_96_Nb_4_ Bulk Glassy Alloys. J. Appl. Phys..

[52] Shen B., Inoue A. (2005). (Fe,Co,Ni)–B–Si–Nb Bulk Glassy Alloy With Super-High Strength and Some Ductility [Article Retracted]. J. Mater. Res..

[53] Pang L.L., Inoue A., Zanaeva E.N., Wang F., Bazlov A.I., Han Y., Kong F.L., Zhu S.L., Shull R.B. (2019). Nanocrystallization, Good Soft Magnetic Properties and Ultrahigh Mechanical Strength for Fe_82–85_B_13–16_Si_1_Cu_1_ Amorphous Alloys. J. Alloy Compd..

[54] Han J., Wang C., Kou S., Liu X. (2013). Thermal Stability, Crystallization Behavior, Vickers Hardness and Magnetic Properties of Fe-Co-Ni-Cr-Mo-C-B-Y Bulk Metallic Glasses. Trans. Nonferrous Met. Soc. China.

[55] Błoch K., Nabiałek M., Postawa P., Sandu A.V., Śliwa A., Jeż B. (2020). The Magnetisation Process of Bulk Amorphous Alloys: Fe_36+x_Co_36−x_Y_8_B_20_, Where: X = 0, 3, 7, or 12. Materials.

[56] Nabiałek M., Jeż B., Pietrusiewicz P., Jeż K., Płoszaj B., Sandu A.V., Abdullah M.M.A.B., Wysłocki J., Kalwik A., Postawa P. (2021). Effect of Chemical Composition on Curie Temperature of FeCoB Alloys. Acta Phys. Pol. A.

[57] Olekšáková D., Kollár P., Füzer J., Onderko F., Dobák S., Viňáš J., Fáberová M., Bureš R. (2017). Magnetic Properties of Sintered Fe_{50}Co_{50} Powder Cores. Acta Phys. Pol. A.

[58] Mohapatra J., Xing M., Elkins J., Liu J.P. (2020). Hard and Semi-Hard Magnetic Materials Based on Cobalt and Cobalt Alloys. J. Alloy Compd..

[59] Nematov M.G., Baraban I., Yudanov N.A., Rodionova V., Qin F.X., Peng H.-X., Panina L.V. (2020). Evolution of the Magnetic Anisotropy and Magnetostriction in Co-Based Amorphous Alloys Microwires Due to Current Annealing and Stress-Sensory Applications. J. Alloy Compd..

[60] Zhukov A., Talaat A., Ipatov M., Blanco J.M., Zhukova V. (2014). Tailoring of Magnetic Properties and GMI Effect of Co-Rich Amorphous Microwires by Heat Treatment. J. Alloy Compd..

